# Peer review trends in six fisheries science journals

**DOI:** 10.1186/s41073-024-00146-8

**Published:** 2024-06-25

**Authors:** Stephen R. Midway, Laura Hendee, Daniel J. Daugherty

**Affiliations:** 1 Department of Oceanography and Coastal Sciences, Louisiana State University, Baton Rouge, LA 70820 USA; 2 American Fisheries Society, 425 Barlow Place, Suite 110, Bethesda, MD 20814 USA; 3 Texas Parks and Wildlife Department, Heart of the Hills Fisheries Science Center, 5103 Junction Highway, Mountain Home, TX 78058 USA

**Keywords:** Reviewer invitations, Time in review, Rejection rate, Double-blinding

## Abstract

**Background:**

As the production of scientific manuscripts and journal options both increase, the peer review process remains at the center of quality control. Recent advances in understanding reviewer biases and behaviors along with electronic manuscript handling records have allowed unprecedented investigations into the peer review process.

**Methods:**

We examined a sample of six journals within the field of fisheries science (and all published by the American Fisheries Society) specifically looking for changes in reviewer invitation rates, review time, patterns of reviewer agreements, and rejection rates relative to different forms of blinding.

**Results:**

Data from 6,606 manuscripts from 2011–2021 showed significant increases in reviewer invitations. Specifically, four journals showed statistically significant increases in reviewer invitations while two showed no change. Review times changed relatively little (± 2 weeks), and we found no concerning patterns in reviewer agreement. However, we documented a consistently higher rejection rate—around 20% higher—of double-blinded manuscripts when compared to single-blinded manuscripts.

**Conclusions:**

Our findings likely represent broader trends across fisheries science publications, and possibly extend to other life science disciplines. Because peer review remains a primary tool for scientific quality control, authors and editors are encouraged to understand the process and evaluate its performance at whatever level can help in the creation of trusted science. Minimally, our findings can help the six journals we investigated to better understand and improve their peer review processes.

**Supplementary Information:**

The online version contains supplementary material available at 10.1186/s41073-024-00146-8.

## Background

Peer review is a widely recognized scientific evaluation process that may date back hundreds of years but has modern roots in the 1960s [1, 2]. Recent valuations of peer review have calculated that in 2020 reviewers worldwide dedicated >100 million hours; for U.S. reviewers, this was equal to $1.5 billion in peer review effort [3]. The advent of the internet has also been a boon to publishing [4] as submissions and reviews are now transferred electronically, resulting in faster handling and review times. In terms of performance, peer review can work extremely well when objective experts come together to evaluate (and hopefully improve) scientific works before they are shared and subsequently built on. However, when the process loses rigor, peer review may do little to improve a scientific work—and at worst, a failed peer review process may allow erroneous or biased science to enter the public domain. Because peer review is essentially a self-regulating system that science depends on to produce quality work, scientists and publishers should regularly engage in auditing the peer review process to ensure it remains efficient, effective, and continues to serve its intended role. Specifically, the last few years have shed light on several challenges to the peer review system, including peer reviewer biases [5], high variability among reviews [6], effects of the COVID-19 pandemic [7], the role blinding (also referred to as *anonymized*) plays in publication outcomes [8], and open peer review, a model of open identity, open reports, and open participation in peer review [9].

Although we tend to think about the scientific implications of peer review, there is a strong psychological and sociological aspect of reviewing that may be as simple as understanding what motivates reviewers to undertake reviews. Petchey, Fox and Haddon [10] looked at the balance between authorships and reviews by individual researchers, concluding that, in aggregate, authors are unbalanced in terms of their submissions and reviews to the scientific process, with up to half of authors possibly submitting fewer reviews than manuscripts. Material rewards, such as payment, have been tested as a motivator for peer reviewers; however, review quality and efficiency were found to decrease with rewards [11] and simple reciprocity motives remain a strong factor in taking on and completing reviews. Although there are more scientists submitting more manuscripts than ever [12], reviewing remains a voluntary activity that is often a lower priority than other tasks that directly contribute to job promotion, tenure, or other career advancements [13]. Perhaps unsurprisingly, Mrowinski et al. [14] found that completion rates of reviews were highest when the reviewers knew the editor. Professional relationships can be helpful when seeking reviews, yet biases like homophily [15] also need to be considered so that individuals and groups are not excluded from the review process and manuscripts face a diversity of comments that should ultimately improve the science. Although there is not one solution to biases in the peer review system, authors, editors, and publishers need to be aware that biases are omnipresent [5, 16], whether in a traditional or open peer review system.

On top of existing peer review dynamics, the COVID-19 pandemic (henceforth, *the pandemic*) and its unequal effects have added complexity. Surprisingly, several studies have reported a lack of pandemic effects within journals or fields. Fox and Meyer [17] reviewed data for six ecology journals during the pandemic and found virtually no changes in geographic patterns of submission, submissions from women, and editorial handling time of manuscripts. Some studies have even reported faster review times during the pandemic [7, 18, 19], which may be a result of reviewers having more discretionary time—although these studies also caution about potential for decreased quality in reviews. In contrast, Squazzoni et al. [20] reviewed over 2,000 Elsevier-published journals and found that women submitted proportionally fewer manuscripts than men during the pandemic. In reality, the effect of the pandemic is likely to be both variable and dynamic at different levels (i.e., authors, reviewers, editors) and simple relaxations of review norms (e.g., such as limiting requests for more experiments and unlimited review times; [21]) may be the best approach until effects on specific journals or fields can be identified.

Blinding is a final factor that merits investigation because different forms of blinding may result in different acceptance or rejection rates. Much of science is assumed to be reviewed under a double-blind system, in which both the authors and reviewers do not know each other. However, many disciplines operate as single-blind, in which the reviewers know the author identities. In theory, author identity or affiliation should not factor into an objective decision about scientific quality; however, several studies have found that blinding does play a role in decisions. Specifically, studies have found higher rejection rates (i.e., lower acceptance rates) in double-blind studies [8, 22]. It is thought that single blind reviewers make use of author information, and that prestigious authors and institutes may benefit from being known [23]. Unfortunately, the differential rejection rate from blinding may also have a bias. For example, female first authors were found to benefit from double-blind reviews in *Behavioral Ecology* [22] and blinding male author names was associated with a significant decrease in acceptance [8].

This study was designed to address questions relevant to the peer-review process and subsequent production of published research in the suite of journals published by the American Fisheries Society (AFS). The first question we asked is whether peer-review invitations have significantly increased in the past decade. Based on anecdotal information (e.g., editorial board observations), we hypothesized that the number of peer-review invitations per manuscript has increased in recent years. As part of the first question, we also wanted to know if the pandemic affected peer review invitations, with the hypothesis being that the pandemic created (for many people) unprecedented personal and professional burdens, resulting in increased reviewer invites per manuscript.

Our second question pertained to whether peer review time had changed over the past decade. Although AFS review deadlines are prescribed as 21 days from the date a peer reviewer accepts an invitation, there is a chance that review times could be increasing or decreasing. For example, if peer review invitations are increasing it could mean that reviewers are busier and subsequently that review times have increased. On the other hand, the overall duration of peer review times has likely decreased as a number of journals have decreased their requested review times, and so reviewers may *a priori* expect to do reviews faster now than in the past. As with the first question, we also wanted to evaluate a potential pandemic effect because of the chance that pandemic burdens could influence time in review.

The third question we had was how often peer reviewers are in agreement on a recommendation. Although editorial boards make decisions about submitted manuscripts, often the primary information in that decision is the peer reviews, and it could be useful to know if peer reviews tend to be in agreement with each other or not and what the additional decisions are associated with different levels of agreement. We had no specific hypothesis about agreement, so we approached this as an exploratory question without an expectation of a specific result.

Our last question was whether blinding had an effect on acceptance rate. We had no information (anecdotal or otherwise) to suggest that in the field of fisheries, blinding influences rejection rates. However, studies [8, 22] have shown higher rejection rates in double-blinded reviews, and we wanted to use data from our journals to quantify any effect of blinding.

## Methods

### The data

All peer reviewer data and associated manuscript data came from six AFS journals within the field of fisheries science: *Fisheries*, *Journal of Aquatic and Animal Health* (JAAH), *Marine and Coastal Fisheries: Dynamics, Management, and Ecosystem Science* (MCF; also abbreviated to *Marine and Coastal Fisheries*), *North American Journal of Aquaculture* (NAJA), *North American Journal of Fisheries Management* (NAJFM), and *Transactions of the American Fisheries Society* (TAFS; Tab1e 1). All six journals are currently published by Wiley, and while they may not represent a truly random sample of journals within the field of fisheries science, 1) they are all indexed by Web of Science, and 2) they represent a diversity of subject areas within fisheries science, meaning that they are independent in terms of editorial boards and reviewer recruitment. For all the analyses we conducted, we opted to analyze each journal individually instead of pooling the data. There was certainly a chance that trends and patterns would be consistent across journals, but because the journals operate in different areas of the fisheries science discipline and have unique editorial boards, the possibility existed that the different journals function differently from each other.

In May of 2022, peer review data (referred to as *reports*) for submitted manuscripts was downloaded by the author team from *ScholarOne Manuscripts*, the online submission system used by the journals. We refer to a *submitted manuscript* (or just *manuscript*) as the document submitted by an author team to a journal for peer review and possibly acceptance and publication. A *scientific article* or *paper* refers to the manuscript after it has been through the peer review process and has been accepted. The role of the author team in managing and editing the journals we investigated allowed us access to the reviewer reports, which are otherwise not available to the public. In the reports, a large number of variables are available; however, we did not include any author names, affiliations, or any identifying information that could link a particular manuscript to its authors or a review to its peer reviewers. All six journals were queried from 2011 to 2021, because the ScholarOne system was not used before 2011 and 2021 was the most recent complete year available. For the journal *Fisheries* we restricted our query to article or feature article manuscript types because *Fisheries* publishes a range of different articles including many we did not want to consider in our analysis because certain article types do not undergo peer review (e.g., Essays, Interviews, Forums). For the other five journals we considered all original research manuscripts included; infrequent instances of manuscripts we did not want to consider (e.g., book reviews) were excluded from our analysis during the steps where we removed manuscripts with zero reviewer invitations. In other words, our data included only manuscripts that underwent one or more peer reviews in their first round of review. Additionally, we considered only initial submissions, which corresponds to the first submission distributed for peer review (i.e., no revised manuscripts were included).

### The analysis

#### Changes in peer review invitations

We define a *peer review invitation* (or *invite* or *request*) as simply the request by a journal editorial board member asking an independent scientist (*peer reviewer*) typically unaffiliated with the journal to provide an evaluation of a manuscript. A peer reviewer may accept, decline, or fail to respond to the invitation. To evaluate whether the number of peer review invitations has changed over the last decade, a Poisson generalized linear model (Eq. 1) was used to evaluate any monotonic change in reviewer invites over time:

1 $${\eta }_{i}=\alpha + \beta {x}_{i}$$

where ${\eta }_{i}$ represents $log({\lambda }_{i})$ , which is the log link function of the counts of peer reviewer invitations for manuscript *i* (assumed to be Poisson distributed), $\alpha$ is the intercept parameter, $\beta$ is the slope parameter (estimating the effect of time) and ${x}_{i}$ is the year of submission for manuscript *i*. Each journal was modeled separately in a Bayesian hierarchical framework where the posterior estimate of $\beta$ was the primary parameter of inference. The Bayesian model parameters were given weakly informative normal priors. Three chains ran for 5,000 iterations, with the first 1,000 iterations discarded as burn in and the remaining chains thinned by removing every other iteration. A total of 6,000 Markov chain Monte Carlo (MCMC) samples represented the posterior distribution, and iteration sizes were informed by preliminary model runs that suggested convergence. Models were run in the R package jagsUI [24] within R [25]. We determined a significant effect (i.e., parameter coefficient) as an effect estimate with a 95% credible interval (CI) not overlapping zero, which indicates strong evidence that 0 is an unlikely parameter value. Although other models were considered—such as time series models or generalized additive models—we were only interested in monotonic changes over a relatively short time (11 years) and as such a generalized linear model was determined to be an appropriate approach. An additional analysis evaluated the potential effect of the pandemic on peer reviewer invitations. The data we used for this analysis were the same as used for the overall analysis on change in peer reviewer invites, with the modification that we only included 2018–2021 manuscripts and compared pre-pandemic to pandemic years, which was a categorical (dummy) variable determined by whether the manuscript was submitted before (*x* = 0) of after (*x* = 1) 1 March, 2020 (which we considered the start of the pandemic). We used a Poisson *t*-test fit in a Bayesian framework with priors and MCMC iterations as described above.

#### Changes in time in review

*Peer review time* is the time it takes the peer reviewer to review the manuscript, starting from the date they accept the invitation (not the date the invitation was made) and ending when they submit the review. To quantify any changes in time in review, we further subset the initial submissions data for reviewers that met the criteria of both agreeing to review and having a date at which their review was submitted (indicating it was completed). We had an initial concern about non-independence in review times because multiple peer reviews are nested within a manuscript. There is a potential that within-manuscript variance could be a factor (if manuscript-level factors like length, complexity, and if the associate editor influenced individual manuscript review times). However, we ran an intraclass correlation (ICC) analysis and found no such effect; the ICC estimate for time in review with a random intercept of *manuscript* was 0.08. The lack of within manuscript variance may simply be because effects truly are weak or non-existent, and most manuscripts (91%) have only two or three reviews, which is a relatively small sample size to estimate within-manuscript variance. Because we had no evidence for within-manuscript variance we used the form of equation 1, because our response variable was assumed to be Poisson distributed and we were interested in the potential of a monotonic change over time. The primary change to equation 1 was that the response variable was the number of days in review. Another minor difference was that the observation level for the model is the peer reviewer rather than the manuscript, which was the observation level in the first analysis. The models were also run in a Bayesian framework with the same priors and MCMC settings as described in the subsection *Changes in peer review invitations*. As with the analysis of peer review invitation, we also tested whether the pandemic effected time in review. For this, a Poisson *t*-test modeled time in review (days) against the categorial factor of the pandemic, represented by two levels—before and during the pandemic. With the exception of the response variable, the model and estimation routine were the same as described for the pandemic analysis of peer review invitations.

### Reviewer agreement

Due to the range of different categorical descriptions of reviewer recommendations (e.g., minor revision, major revision, etc.) we simplified recommendation categories to *reject*, *revise*, or *accept* (Table S1). We then subset only manuscripts with two or three peer reviews. Some manuscripts had only one peer reviewer (7%), which did not provide a chance to evaluate agreement. There were manuscripts with four, five, or six peer reviews, but these manuscripts were a small minority of all manuscripts (2%) and were also omitted. Once we had our final list of manuscripts, we enumerated the manuscripts in which all reviewers provided the same recommendation (absolute agreement), as well as the number of manuscripts in which at least one reviewer recommendation differed from another. Finally, these agreement groups were then compared with the editorial decision of *reject*, *revise*, or *accept*. Overall, we approached the question of reviewer agreement without a strong hypothesis or statistical model, but rather in a way that would generate descriptive results to help us understand the degree to which reviewers agree and how that relates to decision outcomes.

### The effect of blinding

In our initial submission data, there were three categories of blinding: *single*, *double*, and *triple*. At the time of submission, authors choose the type of blinding they prefer. Single blinded is characterized by the reviewers (and editors) knowing the author team identity although the authors do not know who the reviewers are. In double blinding, neither the author team nor peer reviewers know each other’s identity (although the editorial team knows both). Triple blind manuscripts are those in which the author team, peer-reviewers, and editorial team are all unknown to each other. Only 0.1% (30 of 28,970) of manuscript submissions were identified as triple-blind, and as such, we removed these from the analysis because we did not have enough sample size to look at patterns by journals or years. Editorial decisions were the same data used in the *Reviewer Agreement* section (Table S1). Finally, we tabulated the editorial decisions by double or single blinded submission type and by journal. We were primarily interested in the reject decision, because the accept decision is virtually never made on initial submissions (<2% of the time) and because the rejection rate is a common journal metric that is meaningful to authors, editorial boards, and other journals. We were able to calculate rejection rates for each of the six journals we investigated but were unable to do so over time because for some combinations of journal and year the number of double-blind submissions was relatively low (<10%). Furthermore, we did not have specific hypotheses about the effect of time on blinded rejections, so this sample size limitation was not a concern. Lastly, *Fisheries* was excluded from this analysis because they had no double-blinded submissions across the 11 years of data.

## Results

The dataset included 32,501 reviewer records (i.e., record of an invitation, regardless of the outcome) for 6,606 manuscripts. The actual number of completed peer reviews was 14,460. NAJFM and TAFS saw about two to three times the manuscript submissions as JAAH, MCF, and NAJA (Table 1). *Fisheries* tended to only have about 20 to 30 peer reviewed manuscript submissions per year, although this was expected because their format publishes a wide range of article types and fewer articles per issue.

**Table 1:** Descriptions of the journals included in this study. The number of articles refers to the total number of articles that went out for peer review between 2011 to 2021. The 2021 IF refers to the most recent impact factor for each journal (recognizing that impact factors are updated on an annual basis)

**Journal**	**Abbreviation**	**Articles**	**2021 IF**	**Topics**
*Fisheries*	*Fisheries*	359	3.5	Broad interest
*Journal of Aquatic and Animal Health*	JAAH	720	2.9	Aquatic organism health
*Marine and Coastal Fisheries*^a^	MCF	550	2.2	Marine fish and systems
*North American Journal of Aquaculture*	NAJA	966	2.0	Intensive and extensive fish culture
*North American Journal of Fisheries Management*	NAJFM	2218	1.7	Fisheries management
*Transactions of the American Fisheries Society*	TAFS	1793	2.2	Biology, ecology, others

^a^The full name is *Marine and Coastal Fisheries: Dynamics, Management, and Ecosystem Science*, although the journal is routinely referred to as just *Marine and Coastal Fisheries*

### Changes in peer review invitations

**Fig. 1:**
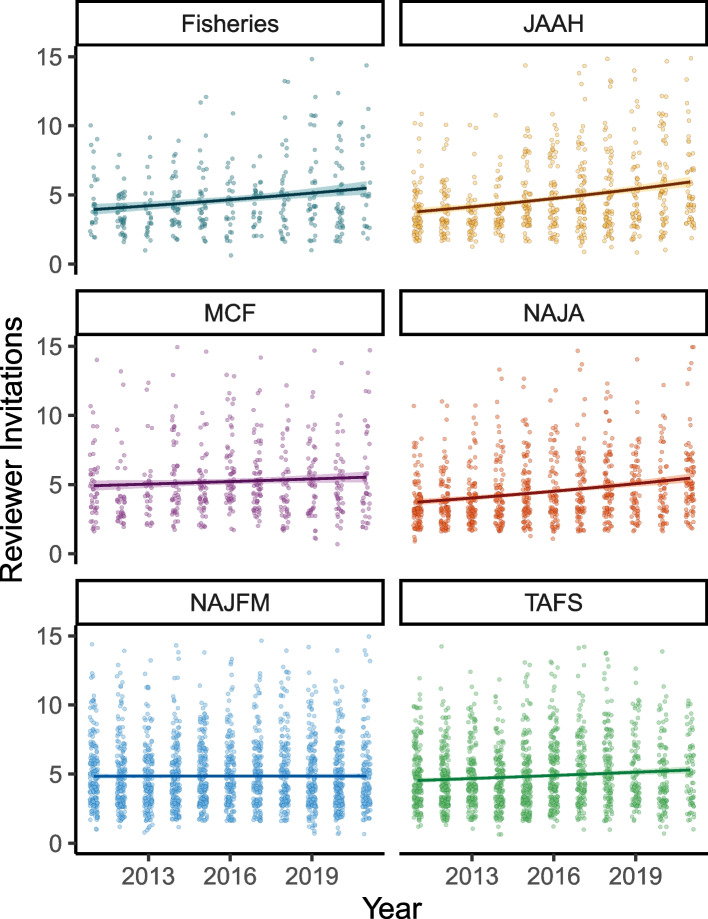
Scatter plots of reviewer invitations from 2011 to 2021 for six fisheries science journals. Individual points represent reviewer invites (jittered to reduce overlap), and the dark line is the Poisson model fit, which was significantly positive for all journals except *Marine and Coastal Fisheries* (MCF) and *North American Journal of Fisheries Management* (NAJFM)

Overall, the number of peer reviewer invitations has generally increased over the past decade. In 2011 the number of peer reviewer invitations per manuscript ranged from 3.7 (NAJA) to 5.3 (MCF); however, in 2021 NAJFM and TAFS had the lowest average number of invites at 5.2, while JAAH had the highest at 7.2 invites per manuscript. Each of the six Poisson GLMs converged, which was based on convergence diagnostics ($\widehat{R}$ < 1.1) and visual inspection of trace plots. MCF and NAJFM were the only two journals that did not see a significant increase in the number of peer review invitations (Table 2; Fig. 1), although neither saw a decrease. Of the four journals that did show a significant increase in peer review invitations, JAAH recorded the greatest increase followed by NAJA, *Fisheries*, and then TAFS (Table 2). The pandemic effect was also journal-specific; NAJA and TAFS showed strong evidence of the pandemic decreasing review invitations, while the pandemic significantly increased review invites for JAAH manuscripts (Table 2). *Fisheries*, MCF, and NAJFM all showed no significant effects (based on the Poisson *t*-test) of any pandemic effects on peer reviewer invites (Table 2).

**Table 2:** For all headers the mean is the posterior mean estimate of time (A and C) or the pandemic (B and D). 2.5% and 97.5% are the lower and upper quantiles, representing the 95% credible interval for the mean. % Same Sign is simply the percentage of MCMC iterations with the same sign as the mean and is just another descriptor of the posterior distribution. Finally, because the mean is reported in terms of the log change of expected counts, the % Effect is a more easily interpreted percent change of the effect. Bold values refer to journals with a significantly positive or negative effect

A) Changes in peer review invites from 2011–2021						
	**Journal**	**Mean**	**2.5%**	**97.5%**	**% Same Sign**	**% Effect**
	*Fisheries*	**0.04**	**0.02**	**0.05**	**100**	**4**
	JAAH	**0.06**	**0.05**	**0.07**	**100**	**7**
	MCF	0.01	-0.01	0.02	79	1
	NAJA	**0.05**	**0.05**	**0.06**	**100**	**6**
	NAJFM	0.00	-0.01	0.01	54	0
	TAFS	**0.02**	**0.01**	**0.03**	**100**	**2**
B) Changes in peer review invites before and during COVID-19 pandemic						
	**Journal**	**Mean**	**2.5%**	**97.5%**	**% Same Sign**	**% Effect**
	*Fisheries*	0.06	-0.08	0.20	80	6
	JAAH	**0.35**	**0.25**	**0.45**	**100**	**42**
	MCF	-0.02	-0.15	0.11	60	-2
	NAJA	**-0.15**	**-0.24**	**-0.05**	**99**	**-14**
	NAJFM	0.04	-0.02	0.11	90	4
	TAFS	-0.08	-0.16	0.01	97	-8
C) Changes in time in review from 2011–2021						
	**Journal**	**Mean**	**2.5%**	**97.5%**	**% Same Sign**	**% Effect**
	*Fisheries*	0.00	0.00	0.01	74	0
	JAAH	0.00	-0.01	0.00	98	0
	MCF	0.00	0.00	0.00	52	0
	NAJA	**-0.02**	**-0.02**	**-0.02**	**100**	**-2**
	NAJFM	**-0.01**	**-0.01**	**-0.01**	**100**	**-1**
	TAFS	**-0.02**	**-0.02**	**-0.01**	**100**	**-2**
D) Changes in time in review before and during COVID-19 pandemic						
	**Journal**	**Mean**	**2.5%**	**97.5%**	**% Same Sign**	**% Effect**
	*Fisheries*	**0.17**	**0.11**	**0.22**	**100**	**18**
	JAAH	**-0.18**	**-0.22**	**-0.14**	**100**	**-17**
	MCF	**-0.10**	**-0.15**	**-0.06**	**100**	**-10**
	NAJA	**-0.06**	**-0.10**	**-0.02**	**99**	**-6**
	NAJFM	-0.02	-0.04	0.01	93	-2
	TAFS	-0.03	-0.06	0.00	99	-3

### Changes in time in review

**Fig. 2:**
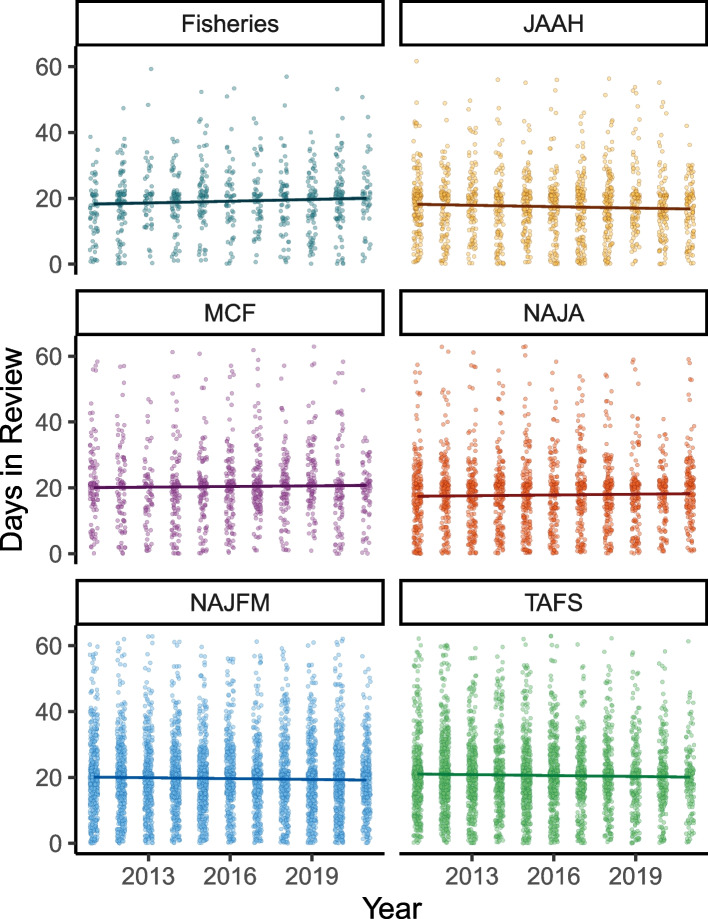
Scatter plots of days in review from 2011 to 2021 for six fisheries science journals. Individual points represent individual reviewers (jittered to reduce overlap) and the dark line is the Poisson model fit, which was significantly negative for *North American Journal of Aquaculture* (NAJA), *North American Journal of Fisheries Management* (NAJFM), and *Transactions of the American Fisheries Society* (TAFS)

The number of days in review tended to either be stable or slightly decrease for some journals. The lowest average number of days in review for any journal in any year was 16 (JAAH in 2021) while the maximum average number of days in review for any journal in any year was 24 days (TAFS in 2012). Overall, most journals had annual averages of days in review around 19–21. Each of the six Poisson GLMs converged, which was based on convergence diagnostics ($\widehat{R}$) and trace plots. *Fisheries*, MCF, and JAAH all showed no evidence for change in time in review over the past decade, and NAJA, NAJFM, and TAFS were all found to have significant decreases in time in review, although the effects were relatively small (Table 2). Interestingly, *Fisheries* manuscripts reviewed during the pandemic had significantly longer times in review than before the pandemic, while JAAH, MCF, and NAJA all saw significantly shorter time in review during the pandemic Fig. 2 (Table 2).

### Reviewer agreement

**Fig. 3:**
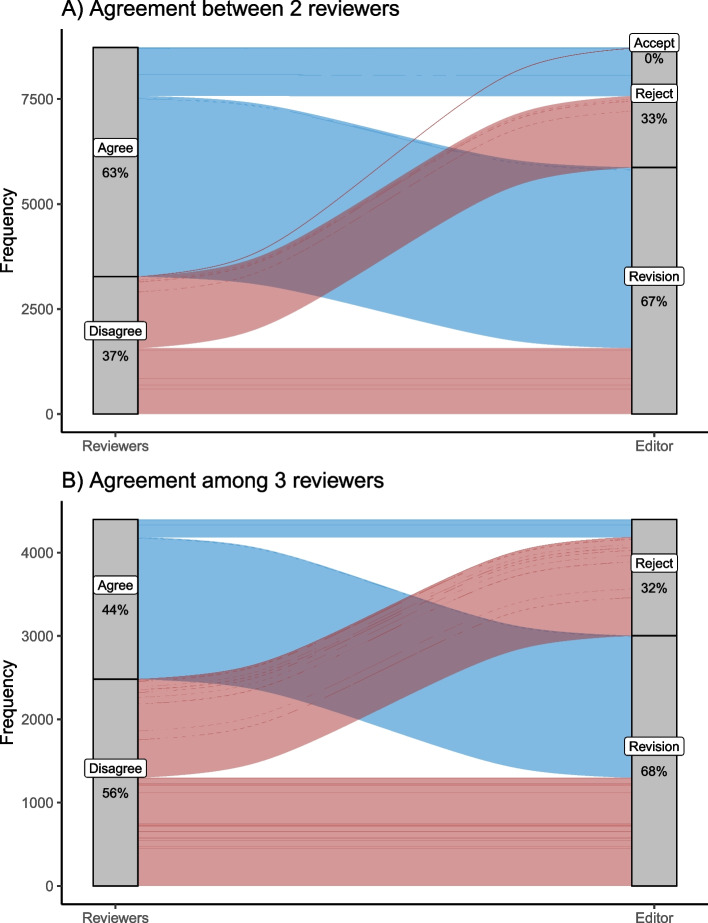
Reviewer agreement for two (**A**) and three (**B**) reviewer scenarios. For each panel the left side represents the reviewer agreement, which is split into the resulting editorial decisions on the left side of each panel. Red bands represent reviewer disagreement and blue bands represent reviewer agreement

For manuscripts with two reviewers, agreement was similar across all six journals, with an overall mean of 63 percent. NAJA had the lowest agreement at 58%, while MCF had the highest agreement at 69%. From manuscripts with three peer reviewers, the agreement dropped to 44% agreement across all reviewers. Percent agreement was again similar among journals ranging from 36% agreement in NAJA to 55% agreement for MCF. Because agreement was similar across all journals, we pooled journals to look at agreement compared to editorial decision. For manuscripts in which two (out of two) reviewers agreed, a majority of the editorial decisions were revision (Fig. 3). Of the 37% of manuscripts in which the two reviewers disagreed, the editorial decisions were roughly equal, with half the manuscripts undergoing revision and half being rejected. The manuscript fates were largely the same under situations with three peer reviewers. Although the overall agreement decreased, the majority of manuscripts under agreement went to revision while the disagreed upon manuscripts were split between revisions and rejection (Fig. 3).

### The effect of blinding

**Fig. 4:**
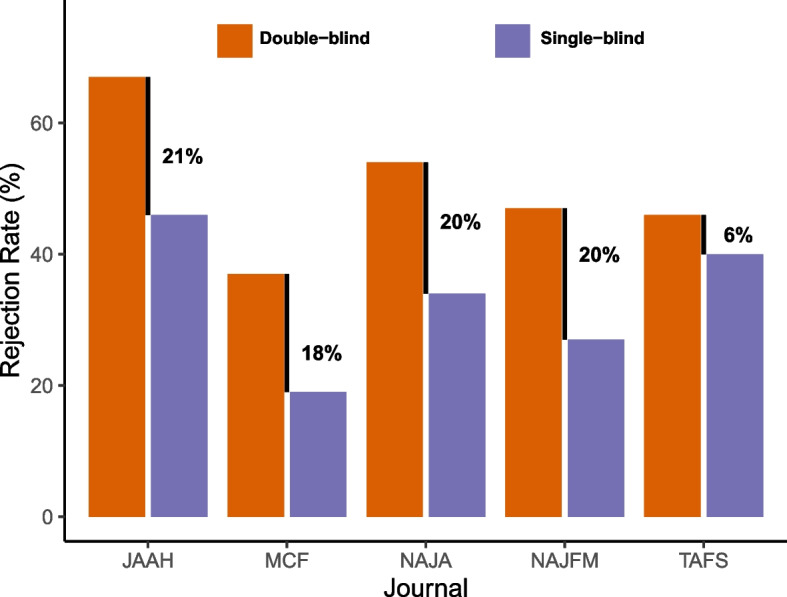
Rejection rates for articles in five fisheries science journals from 2011 to 2021, split by whether the peer review was single or double blinded. The black numbers reporting percentages are the difference within each journal of the single- and double-blind rejection rate

Over 94% of initial submissions (*n* = 26,856) were single-blinded compared to only 1,515 double-blinded submissions. The relatively low number of double-blinded submissions prevented subsetting beyond the journal (e.g., by journal and year): thus, our results are presented by journal. Rejection rates of single-blinded submissions ranged from 19% (MCF) to 46% (JAAH), and all single-blinded rejection rates were lower than double-blinded rejection rates, which were typically around 20% higher than single-blinded rejection rates (Fig. 4). Comparing the two rejection rates within journals, TAFS saw the smallest increase in rejection rate (6%), whereas the double-blind rejection rate for JAAH was 20% greater than the single-blind rejection rate. Although we have reported absolute rejection rates and their differences, we also report that within each journal the relative rejection rates are much higher for double-blinded submissions. The relative rejection rate is the proportional increase compared to a baseline; in our case, the relative increase in double-blind rejection compared to single-blind rejection. Relative rejection rates averaged 58% across journals with a maximum of 95% for MCF (i.e., double-blinded submissions to MCF were rejected almost twice as often as single-blinded submissions).

## Discussion

Our study, which analyzed six fisheries science journals from 2011–2021, found subtle to moderate changes in the peer review process are taking place. Peer review times are not changing across all journals, but most journals are requiring significantly more reviewer invites per manuscript. The effect of the pandemic was variable, but the effect of double blinded manuscripts showed a consistent increase in rejection rates across journals.

The number of peer review invitations increased in four of six journals, and while the numbers may seem relatively small (an average increase of one or two additional reviewers per manuscript over a decade) those one or two additional reviewers are multiplied against the hundreds of manuscripts handled by the journals we studied. Ultimately, the increases we detected may mean that thousands more reviewers will need to be identified and invited to future manuscripts, a trend that has been reported in other disciplines and journals [26]. Overall, the increasing number of invites likely has negative effects on the peer review system. First, additional invitations require additional time for editors to identify peers, invite them to review, and await a response. This added time is directly felt by the authors and indirectly slows down the publication of science. Another concern with increasing invites is the expected quality of the review. Although we have no data to support the idea that reviewer quality decreases with the order of invitation, it is reasonable to question whether secondary, tertiary, or greater invitees, for example, provide the same level of expertise as the primary invitees. To balance these concerns, we note that asking more reviewers may have a positive benefit of including more scientists in the peer review process, and as such it is unknown what the result on scientific quality is when expanded lists of reviewers need to be identified and invited.

As expected, we observed relatively few effects of changing times in review. The expectation of 21 days in review is made clear at the beginning of the review and reinforced with e-mail reminders throughout the review. Additionally, automated reminder emails go out after 21 days to limit additional time in review. However, some journals had slight decreases (around 2%) in review times. Although these decreases were small, they are likely to be accurate given the number of reviews and the number of years we analyzed. We do not know exactly why review times would be slightly decreasing without any clear motive, but it may be attributable to the overall decrease in review times throughout scientific publishing (often led by editorial boards that impose deadlines to stay competitive). Other journals and publishers currently have seven day (Multidisciplinary Digital Publishing Institute [MDPI]) and 14-day review times (Frontiers), and the industry-wide reduction in peer review time may simply be felt across a longer list of journals. Another potential factor assumes that time in review is proportional to effort, which makes it possible that reviews are now shorter and commensurately less effort than in the past. We did not have the data to evaluate review effort or length nor are we hypothesizing that reviewers are putting less effort into reviews—we are merely stating that it is an additional untested explanation for the result we observed and should be considered in future analyses.

Much like the existing literature on the pandemic and peer review, we also observed highly variable pandemic effects. One journal we studied showed a significant increase in reviewer invitations during the pandemic while another journal saw a significant decrease in reviewer invitations. Such variable findings are aligned with what others have reported about the pandemic—both that editorial times were unchanged [17] and review times were faster [7]. Time in review was also mixed with one journal reporting increased review times during the pandemic and three journals reporting decreased review times, but all changes were relatively small. We recognize that analyzing a small number of years before and during the pandemic may not be the optimal way to understand the true effects of the pandemic, especially for variables that change annually without a pandemic effect. But absent any closer investigation of manuscripts or direct surveys of authors, we think that high level comparisons of the time immediately before the pandemic to the time of the pandemic provide at least some insight as to how the pandemic may have changed peer review dynamics.

We did not have well developed questions or hypothesis about reviewer agreement, simply because we had not previously investigated this topic and it is rarely reported in the literature (but see Bornmann [27] for some overview of reviewer agreement). Despite a lack of *a priori* hypotheses, we were unsurprised that about 2/3 of the time two reviewers agreed and a little less than half of the time three reviewers agreed—simply because it is harder to get three people to agree then two people. It is also worth noting that in some cases with two reviewers, a third reviewer may be recruited when there is clear disagreement, and although we cannot quantify this effect it should only lead to an increase in agreement in cases with two reviewers. Agreement heavily favored an editorial decision to revise, as it was relatively rare for two or three reviewers to all agree on a reject. However, the reject outcome was much more likely when reviewers disagreed; roughly half of the disagreements for both two and three reviewer scenarios resulted in an editorial decision to reject. It is difficult for us to make too many inferences from the agreement data simply because we did not have a specific question to answer, but it builds confidence to see that decision outcomes were comparable between two and three reviewers and editorial decisions were not concerningly associated with different agreements. Now that agreement is better quantified, it may create opportunities for future analyses to consider manuscript factors like article length or citations, because a deeper investigation into agreement may inform factors relevant to the manuscripts and not necessarily to the peer reviewers.

Our inferences on the effect of blinding come with the caveat that single blinded manuscripts dominated the data; only about 5% of the initial submissions we looked at were double blinded. Fortunately, our sample sizes were large enough to investigate over 1,500 double-blinded manuscripts, and we saw a consistently higher rejection rate for double-blinded manuscripts compared to single-blinded manuscripts. One journal, TAFS, only had a rejection rate difference of 6% between single and double-blinded reviews, while the four other journals we looked at were all around 20%. Because we did not access any author names, affiliations, or other data it is hard for us to further analyze specific biases of the blinding. But it is possible that we are observing the same biases that other studies have reported.

### Study limitations

We recognize the confounded nature of analyzing the effect of time (in general) on increases in reviewer invites and time in review, while also evaluating the potential effect of the pandemic on the same outcomes. For instance, if a journal showed a significant increase in reviewer invites over the past decade, it could be hard to run a model looking for the effects of the pandemic (which is still essentially evaluating and comparing years) when we know there is an overall trend of time to begin with. We were not able to disentangle the potentially confounding effects of background invitation increases with pandemic effects, effects other than to assume that any potential pandemic effect would be minimal when looking at 11 years of data, and overall long-term effects would be minimal when isolating shorter durations to evaluate the pandemic. And in fact, we did see find journals that showed a long-term increase in reviewer invites while not demonstrating pandemic effects.

Although all journals we analyzed are published by AFS, the journals represent a diverse cross section of fisheries journals. Similarities among the six journals are procedural, such as the AFS standard 21-day review, or the language offered for reviewer recommendations. There are certainly some authors that have published in two or more of the journals we looked at along with some reviewers that may have been invited by multiple journals. But author and reviewer commonality are not expected to be a function of the specific journals we studied, because those authors and reviewers have also likely published in and reviewed for a range of journals outside of those we studied. None of the journals we studied currently has a substantially high or low impact factor (Table 1) that would suggest a hidden effect of prestige (Web of Science 2021 Impact Factors for the Fisheries journal category ranged from 0.3–10.6, whereas the six journals we studied ranged from 1.7–3.5.).

### Recommendations

#### Journal self-evaluation

We do not view this analysis as a detailed audit on peer review in fisheries journals; however, we do think our results provide an interesting and useful look at informative trends for authors and journal editorial staffs. Our analyses illustrate the utility of journal submission data in examining editorial assumptions and quantifying peer-review processes that may benefit from revision. In other words, the better a journal’s editorial board can quantify how peer review operates within their journal, the more control they should have to address problems and shortcomings.

#### Reviewer education

As important as the peer review process is, relatively few peer reviewers ever receive formal training. We encourage current and future reviewers to self-educate using a number of different resources (e.g., [28, 29]). Some journals, particularly those that may be run by scientific societies, may even offer workshops or training on peer review and these can be excellent resources for early reviewers. Lastly, publishers should continue to develop peer review guidance and to invest in promoting these resources in geographic areas or other communities that may be underrepresented in scientific publishing.

### Supplementary Information

Supplementary Material 1. 

Supplementary Material 2. 

## Data Availability

Data for this study is available upon reasonable request to the authors.

## Abbreviations

AFS: American Fisheries Society
JAAH: *Journal of Aquatic and Animal Health*
MCF: *Marine and Coastal Fisheries: Dynamics, Management, and Ecosystem Science*
NAJA: *North American Journal of Aquaculture*
NAJFM: *North American Journal of Fisheries Management*
TAFS: *Transactions of the American Fisheries Society*
MCMC: Markov chain Monte Carlo
ICC: intraclass correlation
GLM: generalized linear model
MDPI: Multidisciplinary Digital Publishing Institute
