# Supplementary material for: Peer review trends in six fisheries science journals
Source: Res Integr Peer Rev. 2024 Jun 25;9:7. doi: 10.1186/s41073-024-00146-8 (PMC11197202; doi:10.1186/s41073-024-00146-8)
Supplement: Supplementary file 1 — Supplementary Material 1. [file 41073_2024_146_MOESM1_ESM.docx]

**Table S1**.

| **Decision** | **Reviewer Language** |
| --- | --- |
| Revision | Major Revision and Resubmit |
|  | Major Revision |
|  | Minor Revision |
|  | Reconsider following revision based on review team comments |
|  | Reconsider following revision. This manuscript should be re-evaluated for acceptance after consideration of the changes suggested by the review team |
|  | Reconsider following revision based on review team comments |
| Reject | Reject from further consideration |
|  | Reject from further consideration |
|  | Reject. The manuscript fails to provide worthwhile information and/or is technically flawed |
|  | Reject and transfer. This manuscript does not fit the aims and scope of the journal and is more appropriate for submission to a companion AFS journal |
|  | Reject and resubmit. Revision of this manuscript will result in substantial changes to the text, analyses, and interpretation of the data that are most consistent with submission as a new manuscript |
| Accept | Publish as-is (i.e., no revision is necessary) |
|  | Accept as-is. This manuscript should be accepted as written and proceed to copy editing and page proofing by the publisher |
