# Supplementary material for: Peer review trends in six fisheries science journals
Source: Res Integr Peer Rev. 2024 Jun 25;9:7. doi: 10.1186/s41073-024-00146-8 (PMC11197202; doi:10.1186/s41073-024-00146-8)
Supplement: Supplementary file 2 — Supplementary Material 2. [file 41073_2024_146_MOESM2_ESM.docx]

Online Supplement

Midway SR, Hendee L, Daugherty, D. Peer review trends in fisheries science journals. *Research Integrity and Peer Review* (In Review)

**Table of Contents**

eResults

eTable 1. Reviewer recommendation language…………………………………………….2

eResults

eTable 1. Reviewer recommendation language

Language presented to reviews and corresponding decision term used in this study. The variability in review language is due to different journals adopting different wording, in addition to some journals changing the language over the years of the study.

| Decision | Reviewer Language |
| --- | --- |
| Revision | Major Revision and Resubmit |
|  | Major Revision |
|  | Minor Revision |
|  | Reconsider following revision based on review team comments |
|  | Reconsider following revision. This manuscript should be re-evaluated for acceptance after consideration of the changes suggested by the review team |
|  | Reconsider following revision based on review team comments |
| Reject | Reject from further consideration |
|  | Reject from further consideration |
|  | Reject. The manuscript fails to provide worthwhile information and/or is technically flawed |
|  | Reject and transfer. This manuscript does not fit the aims and scope of the journal and is more appropriate for submission to a companion AFS journal |
|  | Reject and resubmit. Revision of this manuscript will result in substantial changes to the text, analyses, and interpretation of the data that are most consistent with submission as a new manuscript |
| Accept | Publish as-is (i.e., no revision is necessary) |
|  | Accept as-is. This manuscript should be accepted as written and proceed to copy editing and page proofing by the publisher |
| Redirect | Redirect to (please provide suggested alternative outlets in the comment box below) |
| Redirect | Redirect to (please provide suggested alternative outlets below in the comments box) |
